# Supplementary material for: Association between oral microbiome and seven types of cancers in East Asian population: a two-sample Mendelian randomization analysis
Source: Front Mol Biosci. 2023 Nov 21;10:1327893. doi: 10.3389/fmolb.2023.1327893 (PMC10702768; doi:10.3389/fmolb.2023.1327893)
Supplement: Supplementary file 1 [file DataSheet1.ZIP › Supplementary Materials/MR plots of saliva/saliva═╝/colorectal cancer/pheno.907_to_colorectal cancer_leave_one_out.pdf]

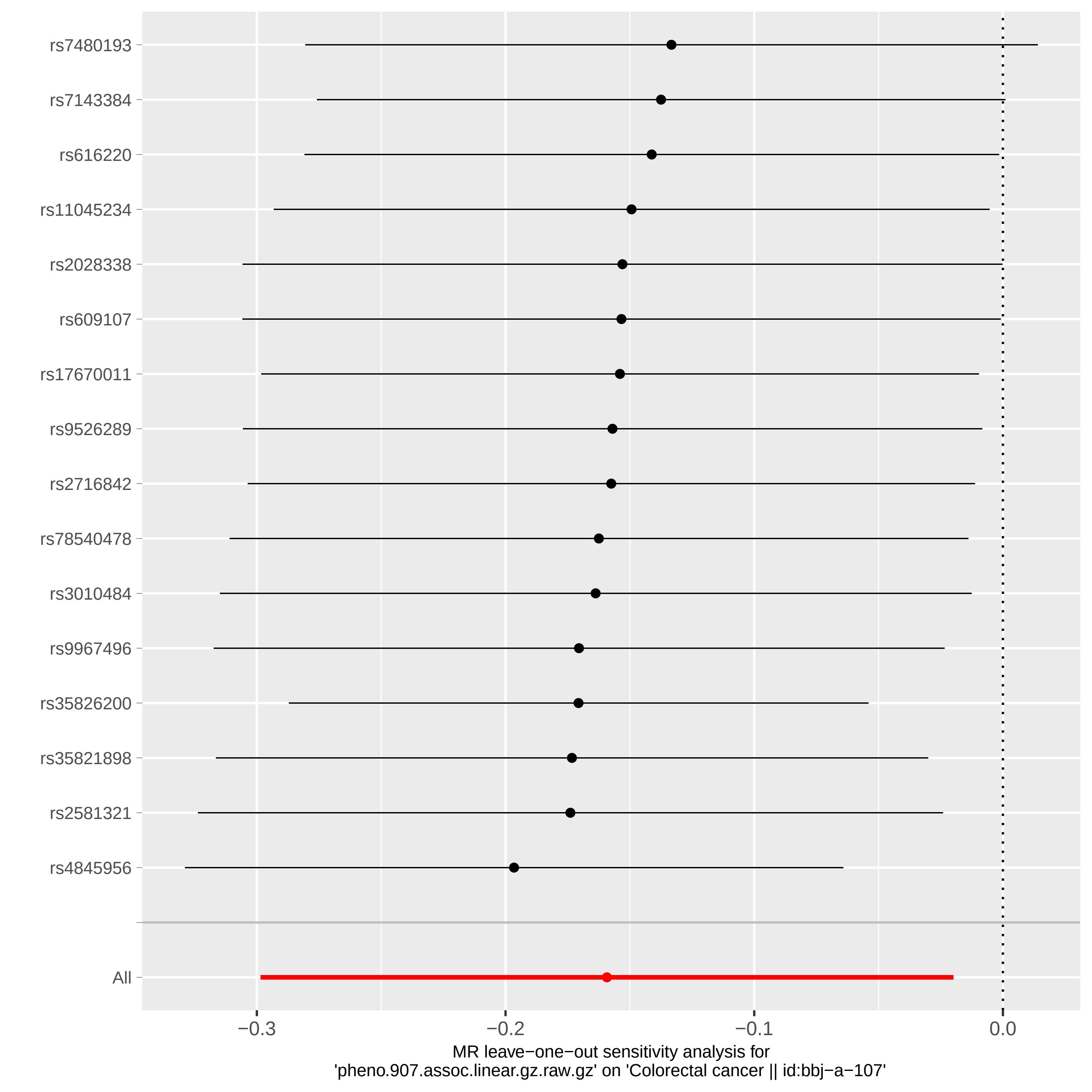
rs7480193
rs7143384
rs616220
rs11045234
rs2028338
rs609107
rs17670011
rs9526289
rs2716842
rs78540478
rs3010484
rs9967496
rs35826200
rs35821898
rs2581321
rs4845956
All
−0.3
−0.2
−0.1
0.0
MR leave−one−out sensitivity analysis for
'pheno.907.assoc.linear.gz.raw.gz' on 'Colorectal cancer || id:bbj−a−107'
